# Dual-Domain Fusion Convolutional Neural Network for Contrast Enhancement Forensics

**DOI:** 10.3390/e23101318

**Published:** 2021-10-09

**Authors:** Pengpeng Yang

**Affiliations:** 1 Institute of Information Science, Beijing Jiaotong University, Beijing 100044, China; ppyang@bjtu.edu.cn; 2 Beijing Key Laboratory of Advanced Information Science and Network Technology, Beijing Jiaotong University, Beijing 100044, China

**Keywords:** contrast-enhanced image detection, pixel-domain, histogram-domain

## Abstract

Contrast enhancement forensics techniques have always been of great interest for the image forensics community, as they can be an effective tool for recovering image history and identifying tampered images. Although several contrast enhancement forensic algorithms have been proposed, their accuracy and robustness against some kinds of processing are still unsatisfactory. In order to attenuate such deficiency, in this paper, we propose a new framework based on dual-domain fusion convolutional neural network to fuse the features of pixel and histogram domains for contrast enhancement forensics. Specifically, we first present a pixel-domain convolutional neural network to automatically capture the patterns of contrast-enhanced images in the pixel domain. Then, we present a histogram-domain convolutional neural network to extract the features in the histogram domain. The feature representations of pixel and histogram domains are fused and fed into two fully connected layers for the classification of contrast-enhanced images. Experimental results show that the proposed method achieves better performance and is robust against pre-JPEG compression and antiforensics attacks, obtaining over 99% detection accuracy for JPEG-compressed images with different QFs and antiforensics attack. In addition, a strategy for performance improvements of CNN-based forensics is explored, which could provide guidance for the design of CNN-based forensics tools.

## 1. Introduction

With the development of image editing techniques, manipulating images is becoming an easy task via various software, such as Photoshop, Meitu, etc., and brings a new challenge for the digital image forensics community. In order to verify the authenticity and integrity of a digital image, numerous algorithms [1,2,3,4] have been proposed. One of the most important research topics in the field of digital image forensics is contrast enhancement forensics. Being a simple yet efficient image processing operation, Contrast enhancement (CE) is typically used by malicious image attackers to eliminate inconsistent brightness when generating visually imperceptible tampered images. CE detection algorithms play an important role in decision analysis of the authenticity and integrity of digital images. Although some schemes have been proposed to detect contrast-enhanced images, the performance of such techniques is limited in the cases of pre-JPEG compression and antiforensic attacks. Therefore, it is critical to develop robust and effective CE forensics algorithms.

Thanks to the efforts of researches in the past decade, a number of schemes [5,6,7,8,9,10,11,12,13] have been proposed to discriminate contrast-enhanced images in an uncompressed format. Stamm et al. [5,6,7] found that contrast enhancement introduced peaks and gaps into the image’s gray-level histogram, which led to specific high values in high-frequency components. Lin et al. [10,11] revealed that contrast enhancement would disturb the interchannel correlation left by color image interpolation and measured such correlation to distinguish the enhanced images from the original images. Furthermore, in order to recover the image processing history, many algorithms for estimating parameters for contrast-enhanced images have been developed [14,15,16,17].

Despite the good performance obtained by the abovementioned algorithms, their robustness can be unsatisfactory in some cases, such as the CE of JPEG images (pre-JPEG compression) and the occurrence of antiforensic attacks [18,19,20,21,22,23]. The reason lies in the fact that the fingerprint left by CE operation would be altered. Based on such a phenomenon, some researchers have proposed more robust CE forensic algorithms, which can be divided into two major branches: overcoming pre-JPEG compression [8] and defending against antiforensic attacks [13]. Unfortunately, neither one of these methods is capable of addressing both pre-JPEG compression and antiforensic attacks. To date, there are no satisfactory solutions for these problems.

With the rapid development of deep-learning techniques, and especially convolutional neural networks (CNNs), some researchers have recently attempted to use them for digital image forensics. A number of preliminary works exploring CNNs in a single domain (such as the pixel domain [24], the histogram domain [25], and the gray-level co-occurrence matrix (GLCM) [26,27]) have been proposed for CE forensics. According to the report [26], deep-learning-based CE forensic schemes achieved better performance than traditional ones. The schemes mentioned above attempt to deal with the CE forensics task by feeding single-domain information to CNNs. However, each domain has its own advantages and disadvantages. For example, according to our experiments, the CNN working in the pixel domain is robust to postprocessing but hard to obtain satisfactory performance. In addition, it is well-known that histogram domain is effective for CE forensics task but fails to resist CE attacks. Such situations give us a strong incentive to explore fusion algorithm across multiple domains based on deep learning techniques against pre-JPEG compression and antiforensic attacks.

In this paper, we propose a novel framework based on dual-domain fusion convolutional neural network for CE forensics. Specifically, the pixel-domain CNN (P-CNN) is designed for the pattern extraction of contrast-enhanced images in pixel domain. For P-CNN, a high-pass filter is used to reduce the affect of image contents and keep the data distribution balance cooperating with batch normalization [28]. In addition, the histogram-domain CNN (H-CNN) is constructed by feeding a histogram with 256 dimensions into a convolutional neural network. The features obtained from P-CNN and H-CNN are fused together and fed into a classifier with two fully connected layers. Experimental results show that our proposed method outperforms state-of-the-art schemes in the case of uncompressed images and obtains comparable performance in the cases of pre-JPEG compression, antiforensics attack, and CE level variation.

The main contributions of this paper are as follows:

(1) We present a dual-domain fusion framework for CE forensics;

(2) We propose and evaluate two kinds of simple yet effective convolutional neural networks based on pixel and histogram domains;

(3) We explore the design principle of CNN for CE forensics, specifically, by adding preprocessing, improving complexity of the architecture, and selecting a training strategy that includes a fine-tuning technique and data augmentation.

The rest of this paper is organized as follows: Section 2 describes related works in the field of CE forensics. In Section 3, we formulate the problem, and in Section 4, we present the proposed dual-domain fusion CNN framework. In Section 5, experimental results are reported. The conclusion is given in Section 6.

## 2. Related Works

In this section, we provide some descriptions of the related works.

**Figure 1:**
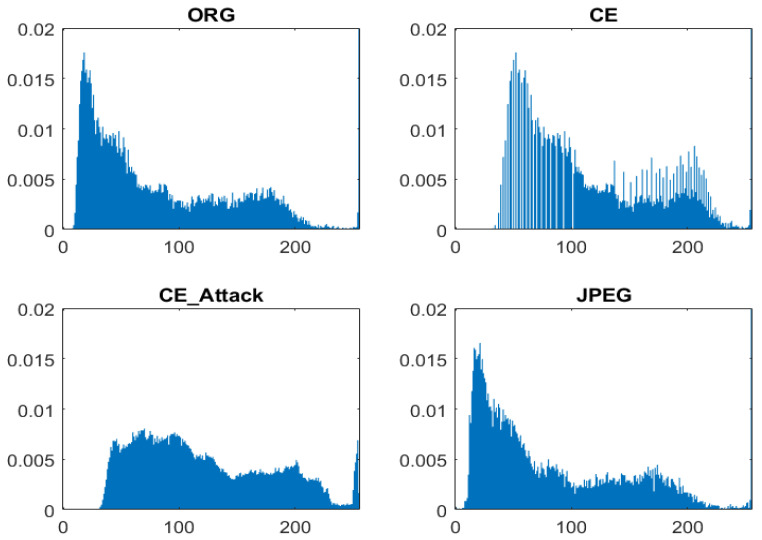
Histogram of uncompressed image, contrast-enhanced image with γ=0.6, contrast-enhanced image in the case of antiforensic attack, and JPEG image with a quality factor equal to 70, respectively.

CE forensics, as a popular topic in the image forensics community, has been studied [1,2] for a long time. Early research works attempted to extract features from the histogram domain. Stamm et al. [5,6,7] observed that the histograms of contrast-enhanced images present peaks/gaps artifacts; in contrast, those of nonenhanced image do not display peaks/gaps, as shown in Figure 1. Based on such observations, they proposed a histogram-based scheme where the high-frequency energy metric is calculated and decided by threshold strategy. However, the above method failed to detect CE images in previously middle/lower-quality JPEG compressed images in which the peak/gap artifacts also exist [8]. Cao et al. [8] studied this issue and found that there exists a notable difference between the peak/gap artifacts from contrast enhancement and those from JPEG compression, which is that the gap bins with zero height always appear in contrast-enhanced images. However, the above phenomenon does not occur in the case of an antiforensics attack. As can be seen in Figure 1, the histogram of an enhanced image with antiforensics attack conforms to a smooth envelope, which is similar with the nonenhanced image.

**Figure 2:**
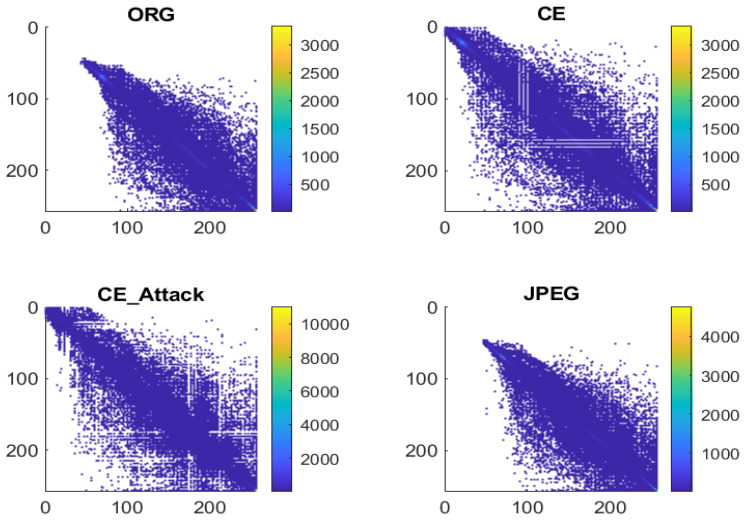
GLCM of uncompressed image, contrast-enhanced image with γ=0.6, contrast-enhanced image in the case of antiforensic attack, and JPEG image with a quality factor is equal to 70.

Instead of exploring the features in histogram domain, De Rosa et al. [13] studied the possibility of using second-order statistics to detect contrast-enhanced images, even in the case of an antiforensics attack. Specifically, the co-occurrence matrix of a gray-level image was explored. According to the report [13], several empty rows and columns appear in the GLCM of contrast-enhanced images, as shown in Figure 2, even after the application of an antiforensics attack [18]. Based on this observation, the authors tried to extract such a feature from the standard deviation of each column of the GLCM. However, its performance is still not satisfactory, especially for the other powerful antiforensics attacks [16].

These algorithms described are based on handcrafted, low-level features, which are not easy to deal with the above problems simultaneously. With the development of data-driven techniques, some researchers have started to study the deep feature representations for CE forensics via data-driven approach using recent and existing methods [24,25,26,27] focused on exploring in single domain. Barni et al. [24] presented a CNN containing a total of nine convolutional layers in the pixel domain, which is similar to the typical CNNs used in the field of computer vision. Cong et al. [25] explored the information in histogram domain and applied the histogram with 256 dimensions into a VGG-based multipath network. Sun et al. [26] proposed calculating the gray-level co-occurrence matrix (GLCM) and feeding it to a CNN with three convolutional layers. Although these approaches based on deep features in single domain have obtained performance gains for CE forensics, they ignore multidomain information, which could be useful in the case that some features in single domain are destroyed.

To overcome the limitation of exiting works, we propose a new deep-learning-based framework to extract and fuse the feature representation in the pixel and histogram domains for CE forensics.

## 3. Problem Formulation

As a common way of contrast enhancement, gamma correction can be found in many image-editing tools. In addition, according to the report [24], enhanced-images with gamma correction are harder to detect than the enhanced images via the other method. Therefore, in this paper, we mainly focus on the detection of gamma correlation, which is typically defined as

(1) $$Y=\left[255{\left(X/255\right)}^{\gamma }\right]\approx 255\left(T^{\gamma }\right)$$

where *X* denotes an input and *Y* represents the remapped value, T=(X/255)ϵ[0,1]. The problem addressed in this paper is how to classify the given image as a contrast-enhanced or nonenhanced image. Particularly, the robustness of the proposed method against pre-JPEG compression and antiforensics attacks is evaluated.

## 4. Proposed Method

In this section, we first present an overview of the proposed framework dual-domain fusion convolutional neural network, and then introduce the major components in detail.

### 4.1. Framework Overview

**Figure 3:**
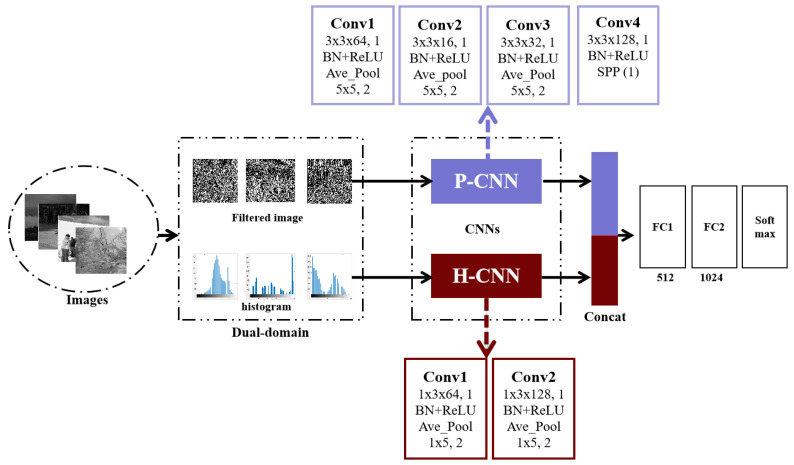
The proposed dual-domain fusion convolutional neural network.

The proposed dual-domain fusion convolutional neural network is shown in Figure 3, which extracts the features from pixel and histogram domains by P-CNN and H-CNN, respectively, and then fuses them before feeding into the classifier with two fully-connected layers. Our end-to-end system predicts whether the image is a contrast-enhanced or nonenhanced image.

### 4.2. Pixel-Domain Convolutional Neural Network

As is well-known, gamma correction leads to the nonlinear changes in pixel domain and introduces peak/gap bins into the histogram domain [5,6,7]. A number of handcrafted features are designed based on such phenomena.

In pixel domain, the difference between the original and enhanced images can be computed as follows, and the absolute value of difference is considered:

(2) $$\left\{\begin{array}{c} D=Y-X=255\left(Z-T\right)\approx 255\left(T^{\gamma }-T\right),\gamma <1\\ D=X-Y=255\left(T-Z\right)\approx 255\left(T-T^{\gamma }\right),\gamma >1 \end{array}\right.$$

It can be seen from (3) that the discriminability in pixel domain is related to the pixel value (image contents) *T* and parameter of gamma correction γ. In order to describe such discriminability, the maximum difference denoted by $D_{max}$ is considered. $D_{max}$ is obtained when the partial derivative of *Z* with respect to *T* is equal to 1.

(3) $$T_{D_{max}}=T_{\frac{\partial Z}{\partial T}=1}={\left(\frac{1}{\gamma }\right)}^{\left(\frac{1}{\gamma -1}\right)}$$

(4) $$D_{max}=\;\left\{\begin{array}{c} 255[{\left(\frac{1}{\gamma }\right)}^{\frac{\gamma }{\gamma -1}}-{\left(\frac{1}{\gamma }\right)}^{\frac{1}{\gamma -1}}],\gamma <1\\ 255[{\left(\frac{1}{\gamma }\right)}^{\frac{1}{\gamma -1}}-{\left(\frac{1}{\gamma }\right)}^{\frac{\gamma }{\gamma -1}}],\gamma >1 \end{array}\right.$$

**Figure 4:**
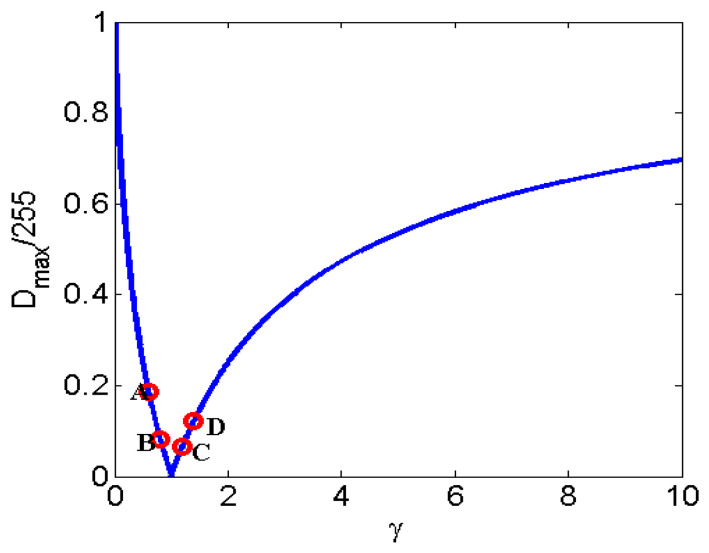
The curve of function of $D_{max}/255$ on γ and A,B,C,D are γ = 0.6, 0.8, 1.2, 1.4, respectively.

The curve of function of $D_{max}/255$ on γ is shown in Figure 4. For the purposes of understanding, four groups of parameters are chosen in the following discussion: γ={0.6,0.8,1.2,1.4}. It is easy to find that $D_{max_{A}}$(γ=0.6)=47.4045 > $D_{max_{D}}$(γ=1.4)=31.416 > $D_{max_{B}}$(γ=0.8)=20.8896 > $D_{max_{C}}$(γ=1.2)=17.0799.

**Figure 5:**
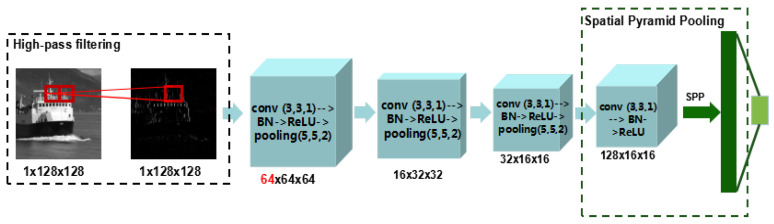
The architecture of proposed pixel-domain convolutional neural networks.

Fortunately, despite the changes in discriminability in the pixel domain, the difference in pixel domain could be learned by deep-learning-based methods. As a popular deep-learning-based technique for image classification, convolutional neural networks (CNNs) in the pixel domain have been applied in image forensics and developed for specific forensic tasks recently. The common modification [29,30] for the CNNs in the forensics community is to add a preprocessing layer that could weaken the effect of image content and improve the signal-to-noise ratio. Inspired by this observation, we performed an experimental study on preprocessing and found an effective CE forensics method (Section 5.3.1). Due to the hardware limitations, we designed a simple 4-layer CNN to keep the balance between performance and computational complexity. The architecture of the proposed pixel-domain convolutional neural network is shown in Figure 5.

Firstly, the high-pass filter is added into the front-end of architecture to eliminate the interference of image content. Another advantage of using a high-pass filter is that it accelerates training by cooperating with batch normalization. The histogram of high-pass filtered images approximately follows the generalized Gaussian distribution, which is similar to batch normalization [28]. In particular, we experimentally found that the filter of the first-order difference along the horizontal direction has better performance.

(5) $$I_{1}=H*I$$

where H=[1,−1], *I* is the input image, $I_{1}$ is the output of the first layer, and ‘∗’ represents the convolution operator.

Next, the high-pass filtering layer is followed by four traditional convolutional layers. For each layer, there are four types of operations: convolution, batch normalization, ReLU, and average pooling. The feature maps for each layer are 64, 16, 32, and 128, respectively. The kernel size for convolutional and pooling operation is 3 × 3 with 1 stride and 5 × 5 with 2 strides. It should be pointed out that (1) we experimentally find that the number of feature maps for the first convolutional layer is important for CE detection, which has better performance when the number of feature maps is 64. In other words, low-level features would be more helpful. (2) Instead of average pooling, the spatial pyramid pooling (SPP) layer [31] is used in the last convolutional layer to fuse multiscale features. The convolutional layer is calculated as

(6) $$I_{i}=\;\left\{\begin{array}{c} P\left(R\left(F\left(W_{i}*I_{i-1}+B_{i}\right)\right)\right),i\epsilon \left(2,3,4\right)\\ S(R\left(F\left(W_{i}*I_{i-1}+B_{i}\right)\right)),i=5 \end{array}\right.$$

where F,R,P,S represent the batch normalization, ReLU, average pooling, and spatial pyramid pooling, respectively. For spatial pyramid pooling, three scales are chosen and lead to 2688 dimensional outputs.

In the end, the fully connected layer and softmax are followed by a multinomial logistic loss. The loss function is defined as

(7) $$Loss=-log(\frac{e^{W^{j}I_{5}+B^{j}}}{\sum_{j=1}^{n}e^{W^{j}I_{5}+B^{j}}})$$

where *n* is the number of classes and *j* denotes the true label. In our experimental setup, Mini-batch Stochastic Gradient Descent is applied and the batch size is set as 120. The learning rate is initialized as 0.001 and scheduled to decrease 10% for every 10,000 iterations. The max iterations is 100,000. Momentum and weight_decay are fixed to 0.9 and 0.0005, respectively.

### 4.3. Histogram-Domain Convolutional Neural Network

According to the report [8], the handcrafted feature based on histogram is also vulnerable. The peak and gap feature is easily destroyed by pre-JPEG compression and antiforensic attacks. In order to detect the CE of JPEG-compressed images, Cao et al. [8] only used the numbers of gap bins as features. However, its performance for different gamma parameters is unstable and it does not work for antiforensics attacks, which could be caused by the unsteadiness of gap bins.

The reason why gamma correction could cause gap bins is that a narrow range of values is projected to a wide one. For example, the values in the range $\left[0,T_{D_{\max}}^{}\right]$, γ<1 will be changed to the range of $\;[0,T_{D_{max}}^{\gamma }]$. Therefore, the probability of gap bins (zero bins) should be proportional to the ratio of the wide range of values and the corresponding strait range,

(8) $$\begin{array}{c} P_{zero\_bin}\propto G\left(r\right)\;=\;\left\{\begin{array}{c} \frac{T_{D_{max}}^{\gamma }\;-\;T_{D_{max}}}{T_{D_{max}}}=\frac{1}{\gamma }-1,\gamma <1\\ \frac{T_{D_{max}}\;-\;T_{D_{max}}^{\gamma }}{1\;-\;T_{D_{max}}}=\frac{{\left(\frac{1}{\gamma }\right)}^{\frac{1}{\gamma -1}}\;-\;{\left(\frac{1}{\gamma }\right)}^{\frac{\gamma }{\gamma -1}}}{1\;-\;{\left(\frac{1}{\gamma }\right)}^{\frac{1}{\gamma -1}}},\gamma >1 \end{array}\right. \end{array}$$

**Figure 6:**
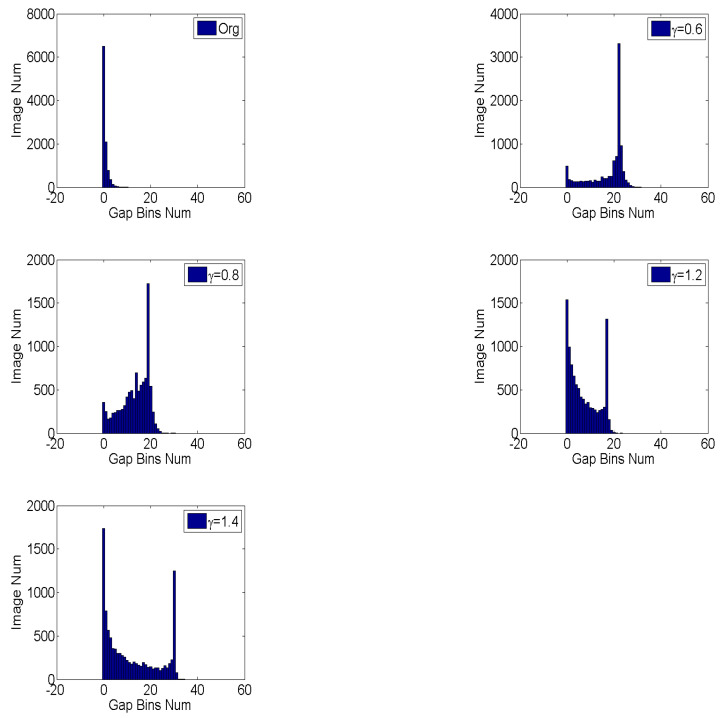
The statistical distribution of gap bins for original and contrast-enhanced images with different parameters. The images are from BOSSBase data-set and centrally cropped into 128 × 128 patches.

**Figure 7:**
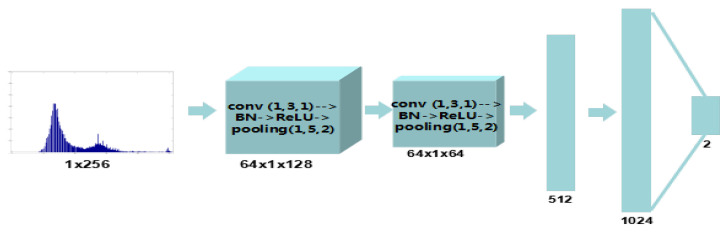
The architecture of the proposed histogram-domain convolutional neural networks.

It can be found that G(0.6)>G(0.8)>G(1.4)>G(1.2), which means that the number of gap bins varies among CE parameters. The statistical distribution of gap bins for the original and enhanced images with γ=0.6,0.8,1.2,1.4 is shown in Figure 6. As can be seen, the numbers of gap bins for γ=0.6,0.8 are larger than γ=1.2,1.4 and the overlapping parts with original images for γ=0.6,0.8 are less than γ=1.2,1.4, which is consistent with the result of our theoretical analysis. Despite the instability of peak/gap bins, we believe that the effective feature could be autolearned from the histogram domain using data-driven algorithm. Instead of designing features, the histogram-domain convolutional neural network is constructed to achieve end-to-end self-learning detection. The H-CNN is proposed to self-learn better features directly from the histogram domain. In addition, as an input with low and fixed dimension, the histogram is suitable for convolutional neural networks. The architecture of H-CNN is shown in Figure 7. Its input is the histogram of the image, namely, a vector with 1 × 256 dimensions. Then, such an input layer is followed by two convolutional and two fully connected layers. The feature maps are 64, 64, 512, and 1024, respectively. Lastly, the softmax layer followed by a multinomial logistic loss, which is added to classify original and enhanced images. The parameters of the convolutional layers and hyperparameters are the same as the P-CNN.

### 4.4. Dual-Domain Fusion Convolutional Neural Network

According to the description of the abovementioned CE forensics, the performance of CE system designed in single domain is still unsatisfactory. Fortunately, fusion strategies [32] provide a good solution to obtain higher performance and have been adopted in the community of digital image forensics [30,33]. In this work, we assume that the features extracted from P-CNN and H-CNN are complementary for CE forensics; thus, we propose a simple yet effective feature fusion framework for deep-learning-based CE forensics to integrate multiple domains and construct the dual-domains fusion CNN (DM-CNN), as shown in Figure 3. Firstly, high-pass filtered images and the histogram are extracted from input images. Then, the filtered images are fed into P-CNN with four 2D-convolutional layers and the histogram is fed into H-CNN with two 1D-convolutional layers. Note that for the purpose of fusion, P-CNN and H-CNN are slightly modified. The P-CNN of DM-CNN is composed of the convolutional layers extracted from the P-CNN. Besides, in order to ensure that the outputs of the P-CNN and H-CNN have the same dimension, one scale of spatial pyramid pooling in P-CNN is chosen and the number of feature maps in the second convolutional layer of H-CNN is set to 128. The features outputs from P-CNN and H-CNN are concatenated together and then fed into classification unit, which consists of two fully connected layers and one softmax layer followed by multinomial logistic loss. It is worth noting that due to the limitation of our hardware configuration, only dual-domains are fused in our system and it would be useful to ensemble features from the other domains.

## 5. Experimental Results

In this section, experimental results are reported. In order to verify the validity of proposed methods, we compared them with four other methods. De Rosa [13], Cao [8], and Sun [26] are proposed for CE forensics. The former two algorithms belong to traditional scheme and the last one is based on deep learning techniques. Li [9] proposed identifying various image operations using high-dimensional, residual-based features. Four groups of experiments are conducted: ORG vs. P-CE, JPEG-ORG vs. JPEG-CE, ORG vs. Anti-CE, and JPEG-ORG vs. JPEG-CE-Anti-CE, where ORG is the original image in an uncompressed format, JPEG-ORG represents original images in JPEG format; P-CE and JPEG-CE denote enhanced versions of ORG and JPEG-ORG, respectively; and Anti-CE and JPEG-CE-Anti-CE represent enhanced images with antiforensics attack for P-CE and JPEG-CE, respectively. The BOSSBase [34] with 10,000 images is chosen to construct the data-set. Firstly, the images are centrally cropped into 128 × 128 pixel patches as ORG. Then, JPEG compression with Q=70,50 is carried out for ORG to build JPEG-ORG. Next, gamma correction with γ={0.6,0.8,1.2,1.4} is implemented on ORG, JPEG-ORG to constitute P-CE and JPEG-CE. In the end, Anti-CE is produced by antiforensics attacks [16,18] on P-CE and JPEG-CE. The reasons for our choice of pixel patch size are as follows: (1) the detection for images with lower resolution is much harder than that with higher resolution images; (2) 128 × 128 is a suitable size for tamper locating based on CE forensics; (3) our hardware configuration is limited. For each experiment, the training, validation, and testing data are 8000, 2000, 10,000, respectively. The experiments about the proposed schemes are conducted on one GPU (NVIDIA TITAN X) with an open-source framework of deep learning: Caffe [35].

### 5.1. Contrast Enhancement Detection: ORG vs. PCE

The results for contrast-enhanced images in an uncompressed format are shown in Table 1. P-CNN is pixel-domain convolutional neural networks and H-CNN is a histogram-domain convolutional neural networks. DM-CNN denotes the dual-domain fusion CNN. As seen in the Table 1, for Cao’s method, the detection accuracy for γ={0.6,0.8} is much higher than that for γ={1.2,1.4}. The reason is that the gap feature is unstable among CE parameters, which is consistent with our analysis in Section III. In addition, H-CNN has better performance than the above four schemes. Such results demonstrated that the histogram domain feature should be effective for CE detection. Besides, the proposed fusion framework—DM-CNN—obtains the best average detection accuracy. It should be mentioned that although the deep-learning-based method proposed by Sun obtained slightly lower detection accuracy than DM-CNN, it has a much higher computational cost during the feature extraction of the GLCM in preprocessing.

### 5.2. Robustness against Pre-JPEG Compressed and Antiforensic Attacked Contrast-Enhanced Images

The performance of different methods for pre-JPEG compressed images with Q={50,70} and antiforensics attacked images are shown in Table 2, Table 3 and Table 4. It can be seen from Table 2 that P-CNN, H-CNN, and DM-CNN have much higher detection accuracy than De Rosa’s and Cao’s methods and have comparable performance with the algorithms proposed by Li and Sun. Besides, there is an interesting phenomenon that the performance of P-CNN has a significant improvement compared with P-CE detection. The reason may be attributed to the fact that JPEG compression weakens the signal components at a high frequency and the difference between original and enhanced images after JPEG compressing would be highlighted.

For antiforensic attacks, Cao’s method does not work and there is a degradation in performance of H-CNN, especially when the antiforensic method [16] is applied. Consequently, the antiforensic attacks would conceal the peak/gap features in histogram domain. In addition, the antiforensics attacks based on histogram may have a slight effect on pixel domain. Therefore, the P-CNN has better performance than H-CNN in this case. When the fusion framework is used to merge pixel and histogram domains together, DM-CNN obtained the best detection accuracy; when the precompression and antiforensic attack are put together, as shown in Table 4, the proposed CNN gains comparable performance with Li’s and Sun’s schemes.

In conclusion, De Rosa’s method is not robust for pre-JPEG compression and antiforensics attack, and Cao’s method is vulnerable to antiforensic attacks. Furthermore, such prior algorithms are unstable in different gamma levels. Although Li’s method based on high-dimensional features is better than previous works in the case of pre-JPEG compression and antiforensic attack, its performance is unsatisfactory when no other operation is used. The deep-learning-based method proposed by Sun obtained slightly lower detection accuracy than the proposed DM-CNN, but it has a much higher computational cost during the feature extraction of the GLCM in preprocessing. Compared with the above schemes, the proposed DM-CNN achieves good robustness against pre-JPEG compression, antiforensic attacks, and CE level variation and obtains the best average detection accuracy in all cases studied.

### 5.3. Exploration on the Strategy to Improve Performance of CNN-Based CE Forensics

Although numerous deep-learning-based schemes have been proposed for digital image forensics, to the best of our knowledge, no one until now has focused on exploring the strategy for performance improvement of single-CNN-based CE forensics. However, it is important for the neophyte to design the new CNN architecture in the community of image forensics. In order to fill such a gap, we make a preliminary exploration in this work. Specifically, there are three parts: adding the preprocessing, improving complexity of architecture, and selecting training strategy, which includes a fine-tuning technique and data augmentation.

#### 5.3.1. Preprocessing

Through protracted and unremitting efforts of researchers, the deep learning technique developed for computer vision (CV) tasks has been succeeded in image forensics. Differing from CV-related tasks, classification in image forensics has little relation to the image content. Therefore, the preprocessing technique has evolved into a universal way to improve the signal-to-noise ratio (SNR). High-pass filtering has become one of most popular means in the preprocessing stage. In this part, using P-CNN in the case of γ=0.6 as an example, we evaluate six kinds of high-pass filters—H1, V1, H2, V2, LAP, HP—that are widely applied in image forensics and compare them with the case without preprocessing. The definitions of these filters are shown in Table 5 and the performance of the above cases is presented in Figure 7. NON means the case without preprocessing. It can be seen that it is not good for CE forensic when non-preprocessing is used. In addition, first-order difference along horizontal direction has better performance. At the same time, the HP and LAP filter proposed for the other forensic task obtained worse performance, which indicates that it is necessary for image forensics to design different high-pass filters.

#### 5.3.2. Powerful Convolutional Neural Networks

**Figure 8:**
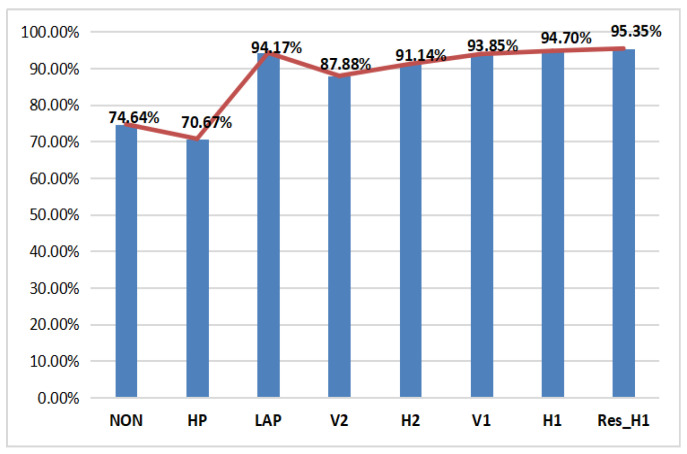
Performance on P-CNN with/without preprocessing and with a powerful network. NON means the case of P-CNN without preprocessing. The others represent the P-CNN with LAP, V2, H2, V1, and H1 filters in the preprocessing. Res_H1 denotes the P-CNN with H1 filter and residual blocks.

Thanks to the development of deep learning techniques in CV, more powerful CNNs (ResNet, XceptionNet, SENet) have surfaced at an increasing rate in recent years. However, because of the limitations in the forensics community, such as insufficient training data-sets and hardware configurations, it would be difficult to evaluate all of them. In order to verify the effectiveness of powerful CNN in CE forensics, based on P-CNN, we replace its traditional convolutional layers with the residual blocks that were proposed in ResNet18 and call it as Res_H1. The result is shown in Figure 8. Compared with the case of H1, detection accuracy of the Res_H1 increases by 0.65%. From the above discussion, we make the conclusion that, for CE forensics, powerful CNNs enhance its performance and preprocessing plays a more important role.

#### 5.3.3. Training Strategy

**Figure 9:**
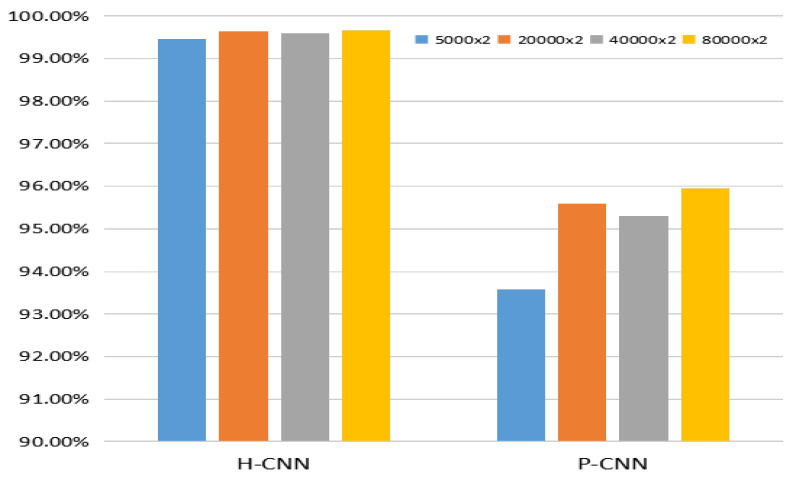
Effect of the scale of training data.

**Figure 10:**
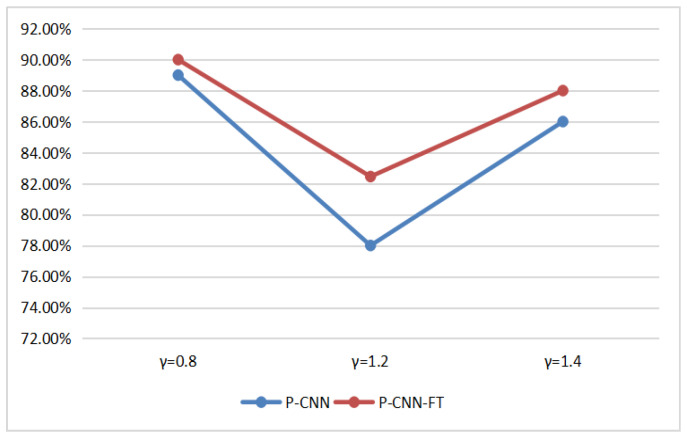
Performance of the P-CNN and the P-CNN with fine-tuning (P-CNN-FT).

It is well-known that the scale of data has an important effect on performance for the deep-learning-based method, and the transfer learning technique [36] also provides an effective strategy to train the CNN model. In this part, we conducted experiments to evaluate the effect of the scale of data and transfer learning strategy on the performance of CNN. For the former, the images from BOSSBase were firstly cropped into 128 × 128 non-overlapping pixel patches. Then, these images were enhanced with γ=0.6. We randomly chose 80,000 image pairs as test data and 5000, 20,000, 40,000, and 80,000 image pairs as training data. Four groups of H-CNN and P-CNN were generated using the above four training data, and the test data is same for these experiments. The result is as shown in Figure 9. It can be seen that the scale of training data has a slight effect on H-CNN with small parameters, and the opposite happens for P-CNN. Therefore, the larger scale of training data is beneficial to the performance of P-CNN with more parameters and the performance of P-CNN would be improved by enlarging the training data. For the latter, we compared the performance of P-CNN with/without transfer learning in the cases of γ={0.8,1.2,1.4}, and the P-CNN with transfer learning by fine-tuning the model for γ={0.8,1.2,1.4} from the model for γ=0.6. As shown in Figure 10, P-CNN-FT achieves better performance than P-CNN.

## 6. Conclusions, Limitations, and Future Research

Being a simple yet efficient image processing operation, CE is typically used by malicious image attackers to eliminate inconsistent brightness when generating visually imperceptible tampered images. CE detection algorithms play an important role in decision analysis for authenticity and integrity of digital images. The existing schemes for contrast enhancement forensics have unsatisfactory performances, especially in the cases of pre-JPEG compression and antiforensic attacks. To deal with such problems, in this paper, a new deep-learning-based framework dual-domain fusion convolutional neural networks (DM-CNN) is proposed. Such a method achieves end-to-end classification based on pixel and histogram domains, which obtain great performance. Experimental results show that our proposed DM-CNN achieves better performance than the state-of-the-art ones and is robust against pre-JPEG compression, antiforensic attacks, and CE level variation. Besides, we explored a strategy to improve the performance of CNN-based CE forensics, which could provide guidance for the design of CNN-based forensics.

In spite of the good performance of exiting schemes, there is a limitation of the proposed method. It is still a hard task to detect CE images in the case of post-JPEG compression with lower-quality factors. The new algorithm should be designed to deal with this problem.

In addition, the security of CNNs has drawn a lot of attention. Therefore, improving the security of CNNs is worth studying in the future.

## Figures and Tables

**Table 1:** CE detection accuracy for contrast-enhanced images in the case that ORG vs. P-CE. AVE is the average accuracy. Best results are marked in bold.

Method	γ=0.6	γ=0.8	γ=1.2	γ=1.4	AVE
De Rosa [13]	94.02%	84.85%	78.37%	74.12%	82.84%
Cao [8]	93.89%	93.90%	80.26%	81.40%	87.36%
Li [9]	93.63%	89.48%	90.76%	93.44%	91.83%
Sun [26]	99.35%	99.21%	98.45%	98.80%	98.95%
P-CNN	94.70%	89.00%	78.00%	86.00%	86.93%
H-CNN	99.48%	99.45%	**99.40%**	99.07%	99.35%
DM-CNN	**99.80%**	**99.72%**	99.36%	**99.41%**	**99.57%**

**Table 2:** CE detection accuracy for pre-JPEG compressed images with different QFs. AVE is the average accuracy. Best results are marked in bold.

QF	Method	γ=0.6	γ=0.8	γ=1.2	γ=1.4	AVE
	De Rosa [13]	81.50%	79.69%	75.16%	72.70%	77.26%
	Cao [8]	93.96%	93.75%	80.36%	81.57%	87.41%
	Li [9]	99.11%	98.59%	97.75%	98.43%	98.47%
50	Sun [26]	99.73%	99.62%	99.40%	99.75%	99.63%
	P-CNN	98.20%	98.25%	96.70%	97.30%	97.61%
	H-CNN	99.90%	99.80%	99.50%	99.78%	99.75%
	DM-CNN	**99.97%**	**99.90%**	**99.86%**	**99.96%**	**99.92%**
	De Rosa [13]	83.99%	82.27%	77.47%	72.95%	80.67%
	Cao [8]	94.06%	93.77%	80.55%	81.56%	87.49%
	Li [9]	98.54%	97.42%	96.22%	97.79%	97.49%
70	Sun [26]	99.32%	99.12%	**99.14%**	98.89%	99.12%
	P-CNN	98.60%	97.00%	95.70%	96.50%	96.95%
	H-CNN	98.86%	99.03%	98.27%	97.68%	98.46%
	DM-CNN	**99.68%**	**99.51%**	99.06%	**99.40%**	**99.41%**

**Table 3:** CE detection accuracy in the case of antiforensics attacks. ‘−’ denotes that the method does not work in this case. AVE is the average accuracy. Best results are marked in bold.

Attack	Method	γ=0.6	γ=0.8	γ=1.2	γ=1.4	AVE
	De Rosa [13]	61.67%	58.83%	55.32%	59.33%	58.79%
	Cao [8]	−	−	−	−	−
	Li [9]	96.30%	95.54%	95.72%	96.55%	96.03%
[16]	Sun [26]	95.53%	89.94%	90.55%	92.42%	92.11%
	P-CNN	**97.90%**	**96.00%**	96.50%	96.55%	96.74%
	H-CNN	88.77%	73.65%	74.85%	78.42%	78.92%
	DM-CNN	97.85%	95.97%	**96.68%**	**97.18%**	**96.92%**
	De Rosa [13]	69.85%	66.03%	62.29%	64.42%	65.65%
	Cao [8]	−	−	−	−	−
	Li [9]	99.57%	99.38%	99.33%	99.51%	99.48%
[18]	Sun [26]	99.48%	99.07%	99.08%	99.19%	99.21%
	P-CNN	98.60%	98.50%	97.80%	98.00%	98.21%
	H-CNN	98.82%	97.59%	97.57%	97.09%	97.77%
	DM-CNN	**99.72%**	**99.78%**	**99.70%**	**99.59%**	**99.70%**

**Table 4:** CE detection accuracy for JPEG-compressed images with different QFs and antiforensics attack [16]. ‘−’ denotes that the method does not work in this case. AVE is the average accuracy. Best results are marked in bold.

QF	Method	γ=0.6	γ=0.8	γ=1.2	γ=1.4	AVE
	De Rosa [13]	70.26%	67.85%	65.38%	66.52%	67.50%
	Cao [8]	−	−	−	−	−
	Li [9]	99.90%	99.90%	99.90%	99.90%	99.90%
50	Sun [26]	99.75%	99.63%	99.68%	99.57%	99.66%
	P-CNN	99.90%	99.90%	99.90%	99.90%	99.90%
	H-CNN	99.45%	99.40%	99.20%	99.20%	99.31%
	DM-CNN	**99.93%**	**99.96%**	**99.97%**	**99.94%**	**99.95%**
	De Rosa [13]	68.68%	65.61%	62.24%	63.93%	65.12%
	Cao [8]	−	−	−	−	−
	Li [9]	99.90%	99.90%	99.90%	**99.90%**	99.90%
70	Sun [26]	99.32%	99.34%	98.60%	99.03%	99.07%
	P-CNN	99.80%	99.75%	99.55%	99.80%	99.73%
	H-CNN	97.35%	98.35%	97.80%	98.15%	97.91%
	DM-CNN	**99.92%**	**99.94%**	**99.95%**	**99.90%**	**99.93%**

**Table 5:** The filters evaluated in this work.

$H1=\;\left[\begin{array}{cc} 1 & -1 \end{array}\right]$	$V1=\;\left[\begin{array}{c} 1\\ -1 \end{array}\right]$	$H2=\;\left[\begin{array}{cc} 1 & 0\\ 0 & -1 \end{array}\right]$
$H2=\;\left[\begin{array}{cc} 0 & -1\\ 1 & 0 \end{array}\right]$	$LAP=\;\left[\begin{array}{ccc} 0 & -1 & 0\\ -1 & 4 & -1\\ 0 & -1 & 0 \end{array}\right]$	$HP=\frac{1}{12}\cdot \left[\begin{array}{ccccc} -1 & 2 & -2 & 2 & -1\\ 2 & -6 & 8 & -6 & 2\\ -2 & 8 & -12 & 8 & -2\\ 2 & -6 & 8 & -6 & 2\\ -1 & 2 & -2 & 2 & -1 \end{array}\right]$

## Data Availability

All the related data is included in the manuscript.

## References

[1] Yang P., Baracchi D., Ni R., Zhao Y., Argenti F., Piva A. (2020). A Survey of Deep Learning-Based Source Image Forensics. J. Imaging.

[2] Camacho I., Wang K. (2021). A Comprehensive Review of Deep-Learning-Based Methods for Image Forensics. J. Imaging.

[3] Chen Y.L., Yau H.T., Yang G.J. (2013). A Maximum Entropy-Based Chaotic Time-Variant Fragile Watermarking Scheme for Image Tampering Detection. Entropy.

[4] Bo Z., Qin G., Liu P. (2015). A Robust Image Tampering Detection Method Based on Maximum Entropy Criteria. Entropy.

[5] Stamm M., Liu K.R. Blind forensics of contrast enhancement in digital images. Proceedings of the 15th IEEE International Conference on Image Processing.

[6] Stamm M.C., Liu K.R. (2010). Forensic detection of image manipulation using statistical intrinsic fingerprints. IEEE Trans. Inf. Forensics Secur..

[7] Stamm M.C., Liu K.R. Forensic estimation and reconstruction of a contrast enhancement mapping. Proceedings of the 2010 IEEE International Conference on Acoustics, Speech and Signal Processing.

[8] Cao G., Zhao Y., Ni R., Li X. (2014). Contrast enhancement-based forensics in digital images. IEEE Trans. Inf. Forensics Secur..

[9] Li H., Luo W., Qiu X., Huang J. (2016). Identification of various image operations using residual-based features. IEEE Trans. Circuits Syst. Video Technol..

[10] Lin X., Li C.T., Hu Y. Exposing image forgery through the detection of contrast enhancement. Proceedings of the 2013 IEEE International Conference on Image Processing.

[11] Lin X., Wei X., Li C.T. Two improved forensic methods of detecting contrast enhancement in digital images. Proceedings of the IS&T/SPIE Electronic Imaging 2014.

[12] Wen L., Qi H., Lyu S. (2018). Contrast enhancement estimation for digital image forensics. ACM Trans. Multimed. Comput. Commun. Appl..

[13] De Rosa A., Fontani M., Massai M., Piva A., Barni M. (2015). Second-order statistics analysis to cope with contrast enhancement counter-forensics. IEEE Signal Process. Lett..

[14] Farid H. (2001). Blind inverse gamma correction. IEEE Trans. Image Process..

[15] Popescu A.C., Farid H. (2004). Statistical tools for digital forensics. Proceedings of the International Workshop on Information Hiding.

[16] Cao G., Zhao Y., Ni R. Forensic estimation of gamma correction in digital images. Proceedings of the 2010 IEEE International Conference on Image Processing.

[17] Wang P., Liu F., Yang C., Luo X. (2018). Parameter estimation of image gamma transformation based on zero-value histogram bin locations. Signal Process. Image Commun..

[18] Barni M., Fontani M., Tondi B. (2012). A universal technique to hide traces of histogram-based image manipulations. Proceedings of the on Multimedia and Security.

[19] Cao G., Zhao Y., Ni R., Tian H. (2010). Anti-forensics of contrast enhancement in digital images. Proceedings of the 12th ACM Workshop on Multimedia and Security.

[20] Kwok C.W., Au O.C., Chui S.H. (2011). Alternative anti-forensics method for contrast enhancement. Proceedings of the International Workshop on Digital Watermarking.

[21] Comesana-Alfaro P., Pérez-González F. Optimal counterforensics for histogram-based forensics. Proceedings of the 2013 IEEE International Conference on Acoustics, Speech and Signal Processing.

[22] Cao G., Zhao Y., Ni R., Tian H., Yu L. (2014). Attacking contrast enhancement forensics in digital images. Sci. China Inf. Sci..

[23] Ravi H., Subramanyam A.V., Emmanuel S. (2015). ACE—An effective anti-forensic contrast enhancement technique. IEEE Signal Process. Lett..

[24] Barni M., Costanzo A., Nowroozi E., Tondi B. CNN-based detection of generic contrast adjustment with jpeg post-processing. Proceedings of the 2018 25th IEEE International Conference on Image Processing (ICIP).

[25] Zhang C., Du D., Ke L., Qi H., Lyu S. Global Contrast Enhancement Detection via Deep Multi-Path Network. Proceedings of the 2018 24th International Conference on Pattern Recognition (ICPR).

[26] Sun J.Y., Kim S.W., Lee S.W., Ko S.J. (2018). A novel contrast enhancement forensics based on convolutional neural networks. Signal Process. Image Commun..

[27] Shan W., Yi Y., Huang R., Xie Y. (2019). Robust contrast enhancement forensics based on convolutional neural networks. Signal Process. Image Commun..

[28] Ioffe S., Szegedy C. (2015). Batch normalization: Accelerating deep network training by reducing internal covariate shift. arXiv.

[29] Yang P., Ni R., Zhao Y. (2016). Recapture image forensics based on Laplacian convolutional neural networks. Proceedings of the International Workshop on Digital Watermarking.

[30] Yang P., Ni R., Zhao Y., Zhao W. (2017). Source camera identification based on content-adaptive fusion residual networks. arXiv.

[31] He K., Zhang X., Ren S., Sun J. (2015). Spatial pyramid pooling in deep convolutional networks for visual recognition. IEEE Trans. Pattern Anal. Mach. Intell..

[32] Mangai U.G., Samanta S., Das S., Chowdhury P.R. (2010). A survey of decision fusion and feature fusion strategies for pattern classification. IETE Tech. Rev..

[33] Fontani M., Bianchi T., De Rosa A., Piva A., Barni M. (2013). A framework for decision fusion in image forensics based on Dempster–Shafer theory of evidence. IEEE Trans. Inf. Forensics Secur..

[34] Stegodata. http://agents.fel.cvut.cz/stegodata/.

[35] Caffe. http://caffe.berkeleyvision.org.

[36] Pan S.J., Yang Q. (2009). A survey on transfer learning. IEEE Trans. Knowl. Data Eng..

